# Stereotypical descriptions of computer science career interests are not representative of many computer scientists

**DOI:** 10.1038/s41598-022-09522-0

**Published:** 2022-04-09

**Authors:** Jenna E. McChesney, Tara S. Behrend, Alexander Glosenberg

**Affiliations:** 1 North Carolina State University, Raleigh, USA; 2 Purdue University, West Lafayette, USA; 3 Loyola Marymount University, Los Angeles, USA

**Keywords:** Psychology, Computer science

## Abstract

Using responses from a large respondent-initiated online survey, we find that the career interests of many current and aspiring computer scientists in the United States diverge from a popular and official depiction of computer scientists’ interests used for career and workforce development worldwide. Distinct profiles of career interests emerged from the data. These profiles suggest that many women in the field value social and artistic expression in a way not currently recognized by established depictions of computer scientists’ interests. Better capturing the diversity of interests in computer science might help to boost women’s, and men’s, engagement in this STEM field.

## Introduction

Women are underrepresented in many occupations devoted to science, technology, engineering, and mathematics (STEM), including in the field of computer science, where women often compose only small shares of many professions^1^. There is an ongoing debate as to why this is the case^2^ and one prominent explanation is that women’s career preferences do not match the characteristics of STEM environments^3^. Both inside and outside of the United States^4^, the empirical basis for characterizing whether various occupations might or might not be a good fit for the preferences of certain people comes from a database established by the United States Department of Labor known as the Occupational Information Network (O*NET)^5^. A strength of O*NET is its collection and use of insights generated from surveying representative samples of actual incumbents across the U.S. in a wide range of occupations. However, there are indications that potentially misleading stereotypes relating to the field of computer science (CS) exist and that such stereotypes might disadvantage women^6,7^. It is possible that such stereotypes are unwittingly reflected in O*NET’s estimations of the interests of those in CS professions. The present study engages with this possibility. Below, we briefly review literature on gender and career preferences, discuss possible gender stereotypes relating to CS occupations, and introduce the development and use of information by O*NET.

### Literature review

Women’s representation in CS is low even in relation to other STEM fields, and reports indicate that this gender gap is not closing fast^1,8^. The career preference view in the literature^2^ would suggest that this gender gap is due, at least in part, to women’s lack of interest in CS professions. It is widely acknowledged that people pursue careers they perceive to be aligned with their personal preferences^9–12^. Moreover, persons who perceive a correspondence between their preferences and the characteristics of their work tend to report higher levels of job satisfaction and performance^13,14^. However, the work preferences of men and women often diverge in predictable ways—as is evidenced by differences among men and women in career interests^15^. Vocational, or career, interests are preferences for certain work activities and work contexts^16^. In particular, it has been observed that men tend to hold interests in work contexts that involve working with inanimate objects (e.g., computers and tools) while women tend to hold interests in working with people^15^. Workforce trends have been used to support this argument. Women tend to be relatively better represented in STEM occupations that emphasize social activities (e.g., medical services and social sciences) but less well-represented in STEM disciplines that emphasize working with non-animate objects, tools and technologies (e.g., computer science and engineering)^3^. However, there is reason to believe that the field of CS has become increasingly diverse in a way that emphasizes social and collaborative realities^17^.

Beyond women’s own preferences, an alternative explanation for women’s underrepresentation is that they might be deterred from CS careers due to stereotypes about the field. Reasoning from social role theory^18^ proposes that individuals make decisions about their desired participation in roles based upon the perceived gendered nature of those roles. If women perceive that CS professions are stereotypically masculine in nature, they might be dissuaded from participating regardless of their career interests. There is evidence regarding gender-based associations in the field of computer science. For example, stereotypes that women are less interested in computer science than men exist^19,20^. In addition, there are gendered stereotypes regarding persons engaged in computer science (e.g., that they are socially isolated) and stereotypes about the nature of computer science work activities (e.g., that such work is non-collaborative in nature)^21^. Providing evidence for the potentially problematic nature of such stereotypes for women, there is evidence that exposure to stereotypical physical environments and role models (e.g., those indicating a focus on video games or science fiction), can have detrimental influences on women’s interest in pursuing computer science occupations^6,22^. There are indications that gender bias is negatively associated with women’s retention in the field. For example, it has been observed that women studying CS in college who considered dropping their major tended to experience gender bias in the classroom^23^.

Importantly, depictions of STEM occupational characteristics and environments, including computer science occupations, are often based on O*NET^3,4,24^. Accurate information about the characteristics of jobs and the workers who fill them is critical for career guidance, reemployment counseling, workforce development, and human resource management^25–27^. To supply such information, O*NET periodically surveys and updates information on over 900 occupations in the U.S. and publishes it in a publicly available database (www.onetonline.org)^26^. While the database characterizes occupations in the U.S., these descriptions are used by individuals, private sector organizations, and governments worldwide^4^. For example, the Australian government has used O*NET data as part of their own workforce and career development efforts. Moreover, the database has been translated for use in European and Central American countries^4^. Indeed, O*NET has been recognized as a leading source of information about work internationally^24^.

One important component of the O*NET database is information regarding the inferred interests of job incumbents in engaging in certain types of work activities or operating in certain work contexts^26^. Such information regarding the assumed interests of job incumbents is often used to assist the career development of adolescents and adults in helping them to choose occupations that might be a good fit for their preferences^27–29^. In addition, such information is believed to facilitate career exploration and development^30^. Such information may also impact women’s retention in the field, as information about the work environment can either strengthen women’s persistence in CS or make them realize they do not want to work in the industry^31^. Indeed, O*NET data on the inferred interests of incumbents in over 1,000 occupations is used in a popular online survey (used by millions of persons) that delivers an estimation of the correspondence between survey-takers’ interests and the inferred interests of incumbents^32^.

As described by O*NET, the inferred career interest ratings are indirectly based on information regarding that occupation, such as the typical work activities performed on the job^33^. Information on work activities is predominately collected through the stratified sampling of workers in the United States^26^. Based on this information, and other information gathered through alternate means, teams of raters make judgments based on these data regarding the appropriateness of interests as defined by the Holland model^34^. This model is composed of six “RIASEC” interest types: Realistic (interest in practical and hands-on tasks), Investigative (interests in analytic/scientific activities), Artistic (interests in creative or self-expressive work), Social (interests in working or communicating with others), Enterprising (interests in selling and/or leading), and Conventional (interests in working with details and/or routines)^34^. Within this model, interests in things tend to correspond most with Realistic interests while Social interests tend to correspond most with an interest in working with people^35^.

### The present study

In light of the ongoing uncertainty regarding the role of preferences and gender stereotypes in shaping women’s representation in CS professions alongside the importance of information on career interests from O*NET in assisting workforce and career development^30^, the present study explores the possibility that, along one or more of the RIASEC dimensions, the inferred interests of those in CS professions might not represent the interests of all persons employed in or aspiring to CS occupations. To accomplish this, we compare overall O*NET estimations of CS interests with responses to a large (over 80,000 responses) and recent (2016) respondent-initiated online survey of career interests.

## Results

To characterize heterogeneity in the interests of persons employed in or aspiring to CS professions, we conducted a latent profile analysis (LPA) of responses to a respondent-initiated online career-interest survey. We conducted two LPAs using samples of 500 survey responses randomly selected from the following two groups: respondents self-reporting that they are unemployed but identifying a CS profession as their “dream job” (herein those aspiring to CS) and respondents self-identifying as employed in a computer science profession (herein referred to as those employed in CS). We were able to replicate our findings using the larger sample (see SI Appendix).

**Figure 1:**
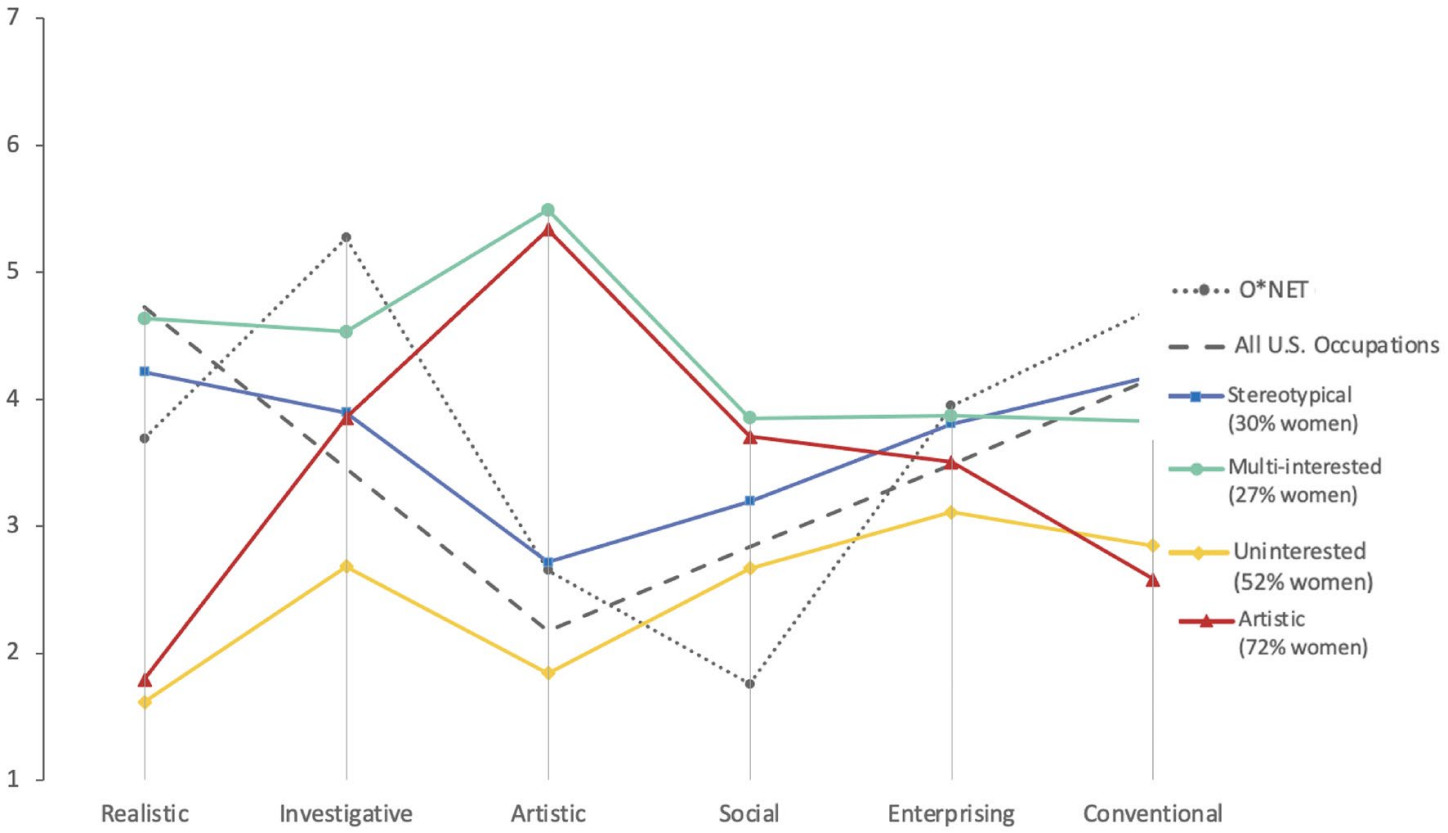
Interest Profiles of Those Employed in CS Compared to O*NET Estimations of CS Interests. Interests were assessed in both the online career interest survey and O*NET according to a scale from 1 to 7, with 7 indicating a stronger preference for/a greater relevance of that interest to the occupation.

**Figure 2:**
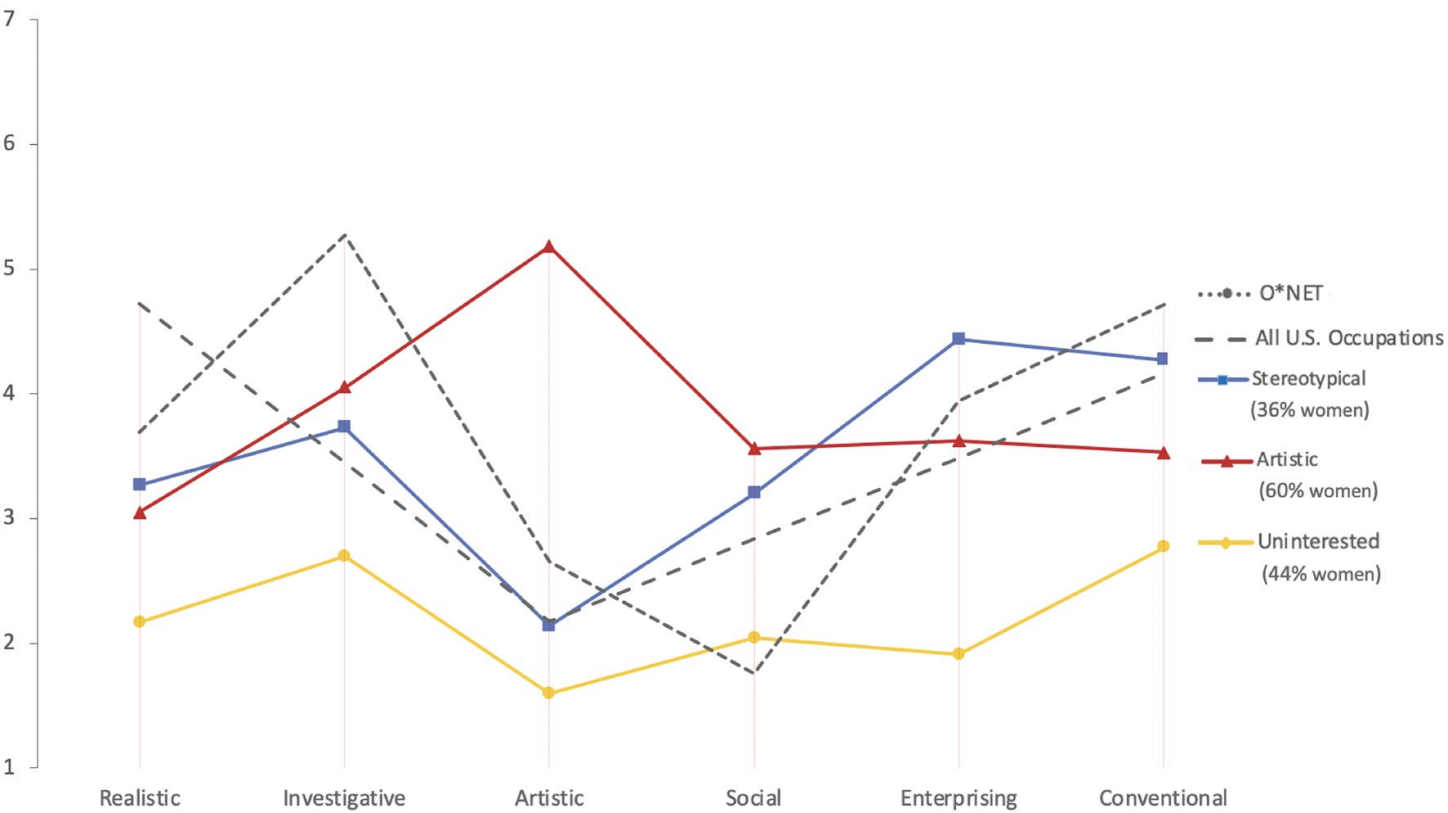
Interest Profiles of Those Aspiring to CS Compared to O*NET Estimations of CS Interests. Interests were assessed in both the online career interest survey and O*NET according to a scale from 1 to 7, with 7 indicating a stronger preference for/a greater relevance of that interest to the occupation.

We found four distinct profiles for those employed in CS (AIC = 9827.48, BIC = 10,029.78) and three profiles for those aspiring to CS (AIC = 10,045.42, BIC = 10,218.22). Descriptive labels and overall characterizations of the structures of those profiles are provided in Table 1. We estimated the inferred interests of those in CS professions using O*NET interests scores by using an average of CS professions—an average weighted by the representation of each profession in our random sample of 1,000 responses. Furthermore, for overall comparative purposes we include average interest scores for the 974 occupations with available interest information from O*NET (herein the U.S. occupational average). Profile scores, the overall O*NET CS interest estimate, and the U.S. occupational average are displayed in Figs. 1 and 2.

**Table 1:** Descriptions of latent profiles.

Sample	Profile	Label	N	% Sample	% Women
Responses among those employed in CS (500 Responses)	1	Artistic	99	20	72
	2	Uninterested	153	31	52
	3	Stereotypical	152	30	30
	4	Multi-interested	96	19	27
Responses among those aspiring to CS (500 Responses)	1	Artistic	204	41	60
	2	Uninterested	115	23	44
	3	Stereotypical	182	36	36

Profiles are listed in descending order of representation for women. See the Online Appendix for values of each profile along all six interest score dimensions and for formalized comparisons of profiles to the O*NET estimate of CS interests.

Profiles roughly resembling the O*NET profile in low estimates of Social and Artistic interests were found among both those employed in CS and those aspiring to CS. We refer to these profiles as “Stereotypical.” As described in further detail in the Supplementary Information, these profiles exhibited a relatively high correspondence to the O*NET estimates in terms of distance scores and correlational comparisons. We note however that the Stereotypical profiles’ low levels of Social and Artistic interests were above the U.S. occupational average. Despite our characterization of these profiles as stereotypical, only 30% (for those employed in CS) and 36% (for those aspiring to CS) of responses fell into these profiles. In contrast, the majority of responses were part of one of three following profiles: Artistic (detected among those employed in and aspiring to CS), Uninterested (detected among those employed in and aspiring to CS), and Multi-Interested (detected only among those employed in CS). The Artistic profiles exhibited high Artistic interests relative to other dimensions. The Multi-Interested profile similarly exhibited high Artistic interests alongside higher Realistic and Investigative interests relative to other profiles. Finally, the Uninterested profile exhibited lower levels of interests than other profiles—except in Conventional interests in comparison to the Artistic profile among those employed in CS. Overall, we note that all seven profiles exhibited higher ratings on the Social interest dimension than the O*NET estimates of CS Social interests. Moreover, Social interest scores for five out of seven profiles were higher than the U.S. occupational average.

Chi-square tests were used to determine whether significant gender differences existed across profiles. Gender was significantly related to profile membership for those employed in CS (χ2(3) = 58.35, p < 0.001) and those aspiring to CS (χ2(2) = 21.65, p < 0.001). We note higher representation of women (72% for those employed in CS/60% for those aspiring to CS) among responses from Artistic profiles and lower representation in Stereotypical profiles (30% for those employed in CS/36% for those aspiring to CS).

## Discussion

To our knowledge, the present study is the largest comparison of O*NET interest profiles with the self-reported interests of actual and aspiring computer scientists in the U.S. A principal finding of this investigation is that the interests of many women do not closely match the interests reflected by influential O*NET depictions of interests in CS. The estimated interests of CS occupations are currently portrayed in O*NET as relatively low on Social dimensions relative to both the latent profile scores and the U.S. occupational average. In contrast, the profile with the highest percentage of women among both those employed in CS occupations and those aspiring to CS occupations is high on Artistic and Social interests relative to the O*NET interest estimate and the U.S. occupational average (Figs. 1 and 2). In relation to Social interests, our results correspond to well-established differences in gendered preferences among men and women with women often expressing stronger interests in working with people^15^. However, we note that the Multi-Interested (Fig. 1) profile among those employed in CS also exhibited relatively high levels of Artistic and Social interests (related to the O*NET and U.S. occupational-average estimates) and that this profile was mostly characterized by men. This result is in alignment with findings that indicate that non-stereotypical depictions of CS professions might be of relevance to both women and men^6^.

Altogether, our results provide nuance to characterizations of CS career interests. Many responses among those in CS professions were characterized by profiles with Social interests that were higher than the U.S. occupational average. This qualifies stereotypical characterizations of CS as occupied by persons preferring social isolation^20^ and supports reasoning that CS is an increasingly diverse field that has grown to engage with the growing social and collaborative nature of computer platforms^17^. We note that it is possible that our results are due to the non-representative sample that we relied upon to characterize CS interests. That is, it is possible that responses in our survey came from individuals engaged in, for example, programming within creative industries and/or in organizational contexts where social interaction is rewarded and/or encouraged. Nevertheless, our results do appear to indicate that a non-trivial share of women and men employed in CS professions do not clearly fit O*NET’s depictions of interests in CS.

The broad pattern observed among those in CS was largely replicated among those aspiring to CS. This is an important finding given that individuals early in their careers or at times when they are considering new career seek out or are often presented with information on occupational interests like that from O*NET to guide their career exploration. Based upon our findings, it might be that those interested in CS with higher Artistic and/or Social interests might be less likely to pursue CS professions after being exposed to characterizations of the CS field as relatively low on Artistic and/or Social interests. While speculative, this would be in alignment with the tendency of people to pursue careers they perceive to be alignment with their personal preferences^9–12^ and would align with research that has found that exposure to stereotypical physical environments and role models can influence women’s interest in computer science occupations^6,21^. Consequently, we call for additional research to explore the potential effects of stereotypical, and non-stereotypical, career interest information on the attitudes of potential CS career prospects. For example, it has been observed that women studying CS in college report fewer positive internship experiences than men, some stating that their internship experiences helped them realize they might want to do something else^23^. Future research could investigate the different types of career-related information provided during CS-related internships and the effects of such information on women’s retention in the field.

The present study focused on the field of CS and future research should explore whether the findings extend to other STEM disciplines. Other STEM fields that are viewed as unsociable include engineering and physics^36^. The male to female ratio among students pursuing college majors in physics, engineering, and computer science have plateaued at about 4:1, and these majors are well known for being underrepresented by women^36^. Future research is needed to understand whether the interest profiles of engineers, physicists, and other STEM fields also deviate from what is conventionally assumed. Such findings could have implications for broadening women’s participation in other STEM fields.

Additional research into the effects of stereotypical and non-stereotypical information might have important practical implications for women’s inclusion in CS professions. There have been a variety of efforts to help qualify stereotypical characterizations of CS. For example, initiatives, like Girls Who Code and INTech Camp for Girls, have sought to help women and girls to successfully enter the CS profession^37,38^. However, such programs often rely on interventions among relatively small populations in certain geographic settings. In contrast, online databases of information about careers, like O*NET, are widely used internationally. Future research should test whether our findings replicate across different countries and cultures. The validity of using O*NET profiles outside of U.S. contexts is questionable given potential variability in work activities internationally^39^. Nevertheless, other English-speaking countries might hold similar occupational stereotypes. For example, the United Kingdom’s National Career Services database (https://nationalcareers.service.gov.uk/) provides occupational descriptions for computing, technology, and digital jobs (e.g., 3D printing technician, software developer, solutions architect). If such job descriptions overemphasize technical aspects of the job (e.g., “write or amend computer code”), this might indicate that social tasks are not as important/relevant to such jobs as they could be in practice.

## Conclusion

Women's lack of representation in CS careers not only denies society the advantage of their abilities and perspectives but also limits their participation in many well paid, high-growth professions^17^. A more nuanced and comprehensive picture of computer scientists’ interests might help address women’s underrepresentation in CS and it is possible that prominent online databases of career information help play a role in this creating this picture.

## Methods

Our characterization of CS interests was drawn from responses to an online survey posted to Time Magazine’s website (see www.time.com; herein the career interest survey). These data were collected in June 2016 and are available from the Open Science Framework (https://bit.ly/3nCBOUN). The first author obtained institutional review board (IRB) approval for the use of archival data generated from this survey for this study (IRB protocol #14,112). As of 2020, the magazine reported having a readership of over 26 million adults, with about 80% of readers living in the United States. Time Magazine’s digital audience is roughly split by gender, with slightly more women (56%) visiting their website than men^40^. The survey was advertised on the magazine’s website as a way for readers to learn more about their career interests and the match of these interests with their occupational aspirations or occupational characteristics. As of December 2021, this survey can be found by going to https://time.com/4343767/job-personality-work/.

Within the survey, respondents were asked to self-declare several demographic characteristics (age, gender, educational attainment, employment status, and personal income). They were then asked to identify their job if they self-identified as being currently employed and identify their aspirational (“dream”) job if they self-identified as unemployed. Finally, they were asked to answer questions about their interests in twenty different types of work activities. At the end of the survey, respondents were given a visual comparison of their interest in different tasks to the characteristics typical of their current or dream job.

### Matching of respondents from the career interest survey to occupations

The occupational title tied to respondent’s job or dream job were calculated using a dynamic keyword search that matched their entered job title in lay terms (e.g., teacher, farmer) with the overarching occupational titles used by O*NET (e.g., elementary school teachers, farmworkers).

### Categorization of CS occupations

We recruited 442 participants from Amazon Mechanical Turk (Mturk) to categorize 707 occupational titles available from the O*NET occupational classification system into one of the following groups: (1) CS (i.e., those requiring some form of experience in computing), (2) STEM (i.e., those requiring some form of experience in science, technology, engineering, and/or mathematics), (3) Non-STEM (i.e., those requiring experience in neither STEM nor computing), or (4) Unsure (i.e., job titles that were unfamiliar with and unsure how to sort). This research was approved by the Institutional Review Board (IRB protocol # 14,159) from North Carolina State University. Informed consent was obtained from all participants, and this research was performed in accordance with the approved protocol and relevant guidelines.

Participants were asked to categorize 80 job titles randomly selected from the overall set of 707 occupational titles. Each job was categorized by 5 to 26 raters, with most jobs assigned 14 raters. Jobs were ultimately classified as CS if more participants classified a job as “CS” than any of the other three categories. Jobs with the same number of participants classifying them as “CS” and another category (e.g., “STEM”) were also classified as CS. The average interrater agreement^41^ for raters’ individual categorization of job titles was strong (rwg = 0.87)^42^. Of the 707 jobs classified, 47 were identified as CS and the remainder were classified as STEM or neither. Of these 47 job titles, 46 were included in the random sample. The 46 job titles included in our final sample (N = 1,000) are provided in Table 2.

**Table 2:** Job titles characterized as CS and counts of responses in random sub-samples.

Job Titles	Count of responses employed in job
Market Research Analysts and Marketing Specialists	109
Software Developers, Applications	88
Graphic Designers	69
Information Technology Project Managers	62
Computer and Information Systems Managers	55
Computer Programmers	50
Operations Research Analysts	45
Computer Systems Analysts	45
Computer and Information Research Scientists	42
Web Developers	37
Computer User Support Specialists	35
Software Developers, Systems Software	31
Network and Computer Systems Administrators	27
Business Intelligence Analysts	25
Computer Hardware Engineers	24
Intelligence Analysts	24
Database Administrators	23
Information Security Analysts	22
Search Marketing Strategists	16
Securities and Commodities Traders	16
Software Quality Assurance Engineers and Testers	14
Database Architects	13
Desktop Publishers	12
Computer, Automated Teller, and Office Machine Repairers	11
Computer Network Architects	10
Data Entry Keyers	10
Quality Control Analysts	9
Video Game Designers	9
Logistics Managers	9
Air Traffic Controllers	9
Sound Engineering Technicians	8
Computer Systems Engineers/Architects	8
Computer Network Support Specialists	7
Audio-Visual and Multimedia Collections Specialists	4
Financial Quantitative Analysts	3
Logistics Analysts	3
Quality Control Systems Managers	3
Computer Science Teachers, Postsecondary	3
Clinical Data Managers	2
Computer Numerically Controlled Machine Tool Programmers, Metal and Plastic	2
Data Warehousing Specialists	1
Robotics Technicians	1
Web Administrators	1
Geographic Information Systems Technicians	1
Microsystems Engineers	1
Gaming Supervisors	1

### Sample

The online survey posted to Time Magazine’s website generated 84,394 responses and we isolated the 40,646 responses to the survey that were provided by those who were 18 years or older and whose internet protocol addresses were from the United States. We eliminated non-U.S. responses due to the questionable comparisons of O*NET data generated in the U.S. to jobs outside of the U.S.^39^. We further isolated 4,662 responses from individuals employed or interested in the 47 occupations that had been categorized as CS using the procedure explained above. Following this, we then randomly selected 500 responses from each of the two groups: (1) those living in the U.S. who self-identified as being employed in CS (4,059 responses) and (2) those living in the U.S. who self-identified as being unemployed and aspiring to CS (603 responses). Responses were randomly selected using the “sample()” function in R (https://www.R-project.org/).

### Employed in CS

As described above, we randomly selected 500 responses from those employed in CS occupations. Of these 500 responses, 56% were from men and 44% were from women. Most responses came from those with an undergraduate (49%) or postgraduate (33%) degree. Only 11% of responses were from individuals between the ages of 18–25 years old, 28% between 26–33 years old, 32% between 34–45 years old, 17% between 46–55 years old, 11% between 56–65 years old, and only 2% were 66 years old or older.

### Aspiring to CS

We also randomly selected 500 responses from those aspiring to CS occupations. Of these 500 responses, 52% were from men and 48% were from women. Most responses came from those with either an undergraduate (43%) or postgraduate (29%) degree. Different ages were also well represented: 26% of responses were from individuals between 18–25 years old, 17% between 26–33 years old, 19% between 34–45 years old, 12% between 46–55 years old, 13% between 56–65 years old, and 12% were 66 years old or older.

### Measures

#### Interests estimated from the online career-interest survey

A shortened version of the popular Personal Globe Inventory (PGI)^43^ was used to assess respondents' career interests. This mini version of the PGI was developed using item response theory^44^ and has been validated for use in the United States by past research^44–46^. Respondents were asked how much they enjoyed 20 different work activities on a scale from 1 (“Strongly dislike”) to 7 (“Strongly like”). Each activity was tied to one of the following six RIASEC career interest types: Realistic, Investigative, Artistic, Social, Enterprising, and Conventional. Like other research using the PGI mini^46^, we utilized item weightings in past research^45^. For each interest type, the corresponding definitions, reliability coefficients, and items are listed below. Reliability was calculated from the overall U.S. sample with Omega total values which provide conservative reliability estimates^45^. Except for the Investigative and Social Scales, Omega total values were at acceptable levels. However, we do not consider these low reliabilities to be problematic. Items composing the scale were selected based upon item response theory analyses of the extent to which a combination of items represented the overall content of each construct. Therefore, in such short scales, low reliabilities can likely be attributed to item’s coverage of different dimensions within the same construct.

- **Realistic** (omega reliability coefficient = 0.78): Involves concrete practical activities and the use of machines, tools, and materials. Realistic interests were measured by asking respondents how much they would enjoy the following tasks: (1) Install electrical wiring and (2) Oversee building construction.
- **Investigative** (omega reliability coefficient = 0.65): Involves analytical or intellectual activity aimed at troubleshooting, creative or knowledge use. Investigative interests were measured by asking respondents how much they would enjoy the following tasks: (1) Categorize different types of wildlife and (2) Write a scientific article.
- **Artistic** (omega reliability coefficient = 0.92): Involves creating work in music, writing, performance, sculpture, or unstructured intellectual endeavors. Artistic interests were measured by asking respondents how much they would enjoy the following tasks: (1) Sculpt a statue, (2) Paint a portrait.
- **Social** (omega reliability coefficient = 0.63): Involves working with others in a helpful or facilitative way. Social interests were measured by asking respondents how much they would enjoy the following tasks: (1) Seat patrons at a restaurant, (2) Interview people for a survey, (3) Help children with learning problems, and (4) Teach people to dance.
- **Enterprising** (omega reliability coefficient = 0.70): Involves selling, leading, and manipulating others to attain personal or organizational goals. Enterprising interests were measured by asking respondents how much they would enjoy the following tasks: (1) Oversee a hotel, (2) Manage an office, (3) Interview people for a survey, and (4) Seat patrons at a restaurant.
- **Conventional** (omega reliability coefficient = 0.85): Involves working with things, numbers, or machines to meet predictable organizational demands or standards. Conventional interests were measured by asking respondents how much they would enjoy the following tasks: (1) Prepare financial reports, (2) Oversee a data analyst group, (3) Maintain office financial records, and (4) Manage an electrical power station.

### Interests estimated from O*NET

As described in greater detail in two technical reports from O*NET on the estimation of interest scores^29,47^, O*NET used two teams of three raters to estimate interest scores for each occupation included in O*NET’s database. These teams reviewed information about each occupations’ tasks, requirements, and generalized work activities to provide RIASEC ratings for over 900 jobs. Some of the information provided to raters (e.g., job’s tasks, requirements, work activities) was gathered by a stratified randomized sampling and surveying of actual job incumbents and occupational experts across the United States^26^.

### Demographic variables

Gender was measured using a dichotomous self-report item, asking whether respondents were “male” (coded as 0 and interpreted as “men”) or “female” (coded as 1 and interpreted as “woman”). Employment status was measured using a single, dichotomous item asking, “Are you currently employed?”. Respondents were also asked to disclose their age and level of educational attainment (less than high school–postgraduate degree).

### Analysis

Profiles were defined solely based on self-reported career interests, which allowed us to determine which profiles naturally emerged for those aspiring to and employed in CS. Model fit indices were used to determine the optimal number of profiles. The number of profiles can differ between samples and analyses, with some models being more parsimonious (having fewer profiles) than others. Similar to previous research^48–51^, we estimated models ranging from two to ten profiles, preferring solutions that did not have classes with a small number of individuals. We used Akogul and Erisoglu’s (2017) analytic hierarchy process (AHP) to select the model with the optimal number of profiles. The AHP takes a holistic approach when considering information criteria, such as Akaike’s Information Criterion (AIC) and Bayesian Information Criterion (BIC), to select the best model. The AHP has been tested on common real and synthetic datasets and found to produce more accurate results than relying on one type of information criteria alone^49^.

### Supplementary Information

Supplementary Information.

## Data Availability

The sample of 84,394 responses from employed and unemployed adults who took the occupational interest survey posted on Time Magazine’s news website are available at https://osf.io/be5ja/. These data are further described in 10.1016/j.jvb.2019.01.002. Data from the sample of 442 adults who categorized 47 job titles as CS (which are listed in Table 2) are available at https://osf.io/f5nrm/?view_only=7b9360268ba14b40a0e051cf3a5020ef

## References

[1] Department of Labor. *Percentage of women workers in science, technology, engineering, and math (STEM).* https://www.dol.gov/agencies/wb/data/occupations-stem (2018).

[2] Kossek EE, Su R, Wu L (2017). "Opting out” or “pushed out”? Integrating perspectives on women’s career equality for gender inclusion and interventions. J. Manag..

[3] Su R, Rounds J (2015). All STEM fields are not created equal: People and things interests explain gender disparities across STEM fields. Front. Psychol..

[4] National Center for O*NET Development. *O*NET products at work*. https://www.onetcenter.org/dl_files/paw/Products_at_Work.pdf (2011).

[5] Peterson NG (2001). Understanding work using the Occupational Information Network (O*NET): Implications for practice and research. Pers. Psychol..

[6] Cheryan S, Plaut VC, Davies PG, Steele CM (2009). Ambient belonging: how stereotypical cues impact gender participation in computer science. J. Pers. Soc. Psychol..

[7] Cheryan S, Siy JO, Vichayapai M, Drury BJ, Kim S (2011). Do female and male role models who embody STEM stereotypes hinder women’s anticipated success in STEM?. Soc. Psychol. Pers. Sci..

[8] Wang LL, Stanovsky G, Weihs L, Etzioni O (2021). Gender trends in computer science authorship. Commun. ACM.

[9] Nye CD, Rounds J (2019). Vocational Interests in the Workplace: Rethinking Behavior at Work.

[10] Strong EK (1943). Vocational Interests of Men and Women.

[11] Van Iddekinge CH, Roth PL, Putka DJ, Lanivich SE (2011). Are you interested? A meta-analysis of relations between vocational interests and employee performance and turnover. J. Appl. Psychol..

[12] Volodina A, Nagy G, Köller O (2015). Success in the first phase of the vocational career: The role of cognitive and scholastic abilities, personality factors, and vocational interests. J. Vocat. Behav..

[13] Hoff KA, Song QC, Wee CJ, Phan WMJ, Rounds J (2020). Interest fit and job satisfaction: A systematic review and meta-analysis. J. Vocat. Behav..

[14] Nye CD, Su R, Rounds J, Drasgow F (2017). Interest congruence and performance: Revisiting recent meta-analytic findings. J. Vocat. Behav..

[15] Su R, Rounds J, Armstrong PI (2009). Men and things, women and people: A meta-analysis of sex differences in interests. Psychol. Bull..

[16] Su R (2020). The three faces of interests: An integrative review of interest research in vocational, organizational, and educational psychology. J. Vocat. Behav..

[17] Brady C, Orton K, Weintrop D, Anton G, Rodriguez S, Wilensky U (2016). All roads lead to computing: Making, participatory simulations, and social computing as pathways to computer science. IEEE Trans. Educ..

[18] Eagly AH, Wood W (2011). Social role theory. Handbook of Theories in Social Psychology.

[19] Master A, Meltzoff AN, Cheryan S (2021). Gender stereotypes about interests start early and cause gender disparities in computer science and engineering. Proc. Natl. Acad. Sci..

[20] Beyer S (2014). Why are women underrepresented in Computer Science? Gender differences in stereotypes, self-efficacy, values, and interests and predictors of future CS course-taking and grades. Comput. Sci. Educ..

[21] Cheryan S, Master A, Meltzoff AN (2015). Cultural stereotypes as gatekeepers: Increasing girls’ interest in computer science and engineering by diversifying stereotypes. Front. Psychol..

[22] Cheryan S (2012). Understanding the paradox in math-related fields: Why do some gender gaps remain while others do not?. Sex Roles.

[23] Kapoor, A., & Gardner-McCune, C. Considerations for switching: Exploring factors behind CS students’ desire to leave a CS major. In *Proceedings of the 23rd Annual ACM Conference on Innovation and Technology in Computer Science Education*, 290–295 (2018).

[24] The International Labour Organization. *Skills for green jobs: A global view*. (eds. Strietska-Ilina, O., Hofmann, C.., Haro, M.D., Jeon, S). 1–155 (2011).

[25] Reiter-Palmon R, Young M, Strange J, Manning R, James J (2006). Occupationally-specific skills: Using skills to define and understand jobs and their requirements. Hum. Resour. Manag. Rev..

[26] Tippins NT, Hilton ML (2010). A Database for a Changing Economy: Review of the Occupational Information Network (O*NET).

[27] Converse PD, Oswald FL, Gillespie MA, Field KA, Bizot EB (2004). Matching individuals to occupations using abilities and the O* NET: Issues and an application in career guidance. Pers. Psychol..

[28] Gore PA, Brown SD (2006). Simpler may still be better: A reply to Eggerth and Andrew. J. Career Assess..

[29] Lara TM, Vess L (2014). Using O*NET in Career Counseling: College Students' Initial Career Choices. Career Plan. Adult Dev. J..

[30] Blustein DL (1992). Applying current theory and research in career exploration to practice. Career Dev. Q..

[31] Pantic K, Clarke-Midura J (2019). Factors that influence retention of women in the computer science major: A systematic literature review. J. Women Minor. Sci. Eng..

[32] Rounds, J., Hoff, K., & Lewis, P. *O*NET interest profiler manual.* National Center for O*NET Development. https://www.onetcenter.org/dl_files/IP_Manual.pdf (2021).

[33] Rounds, J., Armstrong, P., Liao, H. Y., Lewis, P., & Rivkin, D. *Second generation occupational interest profiles for O*NET system: Summary*. National Center for O*NET Development. https://www.onetcenter.org/dl_files/SecondOIP_Summary.pdf (2008).

[34] Holland, J. L. Making vocational choices: A theory of vocational personalities and work environments. In *Psychological Assessment Resources * 3rd edn. (1997).

[35] Tracey, T. J. G., & Sodano, S. M. *Structure of interests and competence perceptions*. In Handbook of vocational psychology: Theory, research, and practice. (Eds. Walsh, W. B., Savickas, M. L., & Hartung, P.) (2013).

[36] Cimpian JR, Kim TH, McDermott ZT (2020). Understanding persistent gender gaps in STEM. Science.

[37] Braswell, K. M. From camp to conferences: Experiences in leveraging tech conferences to inspire black and latinx girls to pursue coding and tech careers. In *2020 Research on Equity and Sustained Participation in Engineering, Computing, and Technology (RESPECT)*, **1**, 1–4 (2020).

[38] Saujani R (2017). Girls Who Code: Learn to Code and Change the World.

[39] Taylor PJ, Li WD, Shi K, Borman WC (2008). The transportability of job information across countries. Pers. Psychol..

[40] Time Magazine. Media Kit-Digital Audience. Retrieved from https://www.timemediakit.com/digital-audience/. (2020).

[41] James LR, Demaree RG, Wolf G (1993). rwg: An assessment of within-group interrater agreement. J. Appl. Psychol..

[42] LeBreton JM, Senter JL (2008). Answers to 20 questions about interrater reliability and interrater agreement. Organ. Res. Methods.

[43] Tracey TJ (2002). Personal globe inventory: Measurement of the spherical model of interests and competence beliefs. J. Vocat. Behav..

[44] Tracey TJ, Tao C (2018). Response latency in interest assessment: An added tool?. J. Vocat. Behav..

[45] Glosenberg A, Tracey TJ, Behrend TS, Blustein DL, Foster LL (2019). Person-vocation fit across the world of work: Evaluating the generalizability of the circular model of vocational interests and social cognitive career theory across 74 countries. J. Vocat. Behav..

[46] Glosenberg, A., Behrend, T., Tracey, T., Blustein, D. L., McChesney, J. E., & Foster, L. Evidence for “pushed out” and “opt out” factors in women’s career inclusion across the world of work in the United States. *J. Career Assess. ***30** (2022).

[47] Lewis, P., & Rivkin, D. *Development of the O* NET interest profiler*. Raleigh, NC: National Center for O* NET Development. (1999).

[48] Perera HN, Mcllveen P (2018). Vocational interest profiles: Profile replicability and relations with the STEM major choice and the Big-Five. J. Vocat. Behav..

[49] Akogul S, Erisoglu M (2017). An approach for determining the number of clusters in a model-based cluster analysis. Entropy.

[50] McLarnon MJ, Carswell JJ, Schneider TJ (2015). A case of mistaken identity? Latent profiles in vocational interests. J. Career Assess..

[51] Perera HN, Calkins C, Part R (2019). Teacher self-efficacy profiles: Determinants, outcomes, and generalizability across teaching level. Contemp. Educ. Psychol..

