# Supplementary material for: Stereotypical descriptions of computer science career interests are not representative of many computer scientists
Source: Sci Rep. 2022 Apr 9;12:5990. doi: 10.1038/s41598-022-09522-0 (PMC8994771; doi:10.1038/s41598-022-09522-0)
Supplement: Supplementary file 1 — Supplementary Information. [file 41598_2022_9522_MOESM1_ESM.docx]

Supporting Information

Stereotypical Descriptions of Computer Science Career Interests Are Not Representative of Many Computer Scientists

Jenna E. McChesney^1^*, Tara S. Behrend^2^, and Alexander Glosenberg^3^

^1^North Carolina State University

^2^Purdue University

^3^Loyola Marymount University

Correspondence: jemcches@ncsu.edu

**Keywords:** gender, occupations, vocational interests, computer science, stereotypes

In addition to the analyses and results presented in the main manuscript, we conducted three sets of supplementary analyses, namely: we formalized comparisons of latent profiles to overall O*NET estimations of CS interests, we made comparisons of latent profile scores to alternative O*NET estimations of CS interests, and we made comparisons of latent profiles found in our sub-samples of 500 responses to profiles drawn from the broader dataset.

**Formalized comparisons between latent profiles and O*NET estimations of CS interests**

Within the main manuscript, we characterized latent profiles with descriptions that in part reflected their relation to the overall estimates of CS interests according to O*NET. Specifically, we identified “Stereotypical” profiles and claimed that these profiles were similar to O*NET estimates of CS interests. To formalize comparisons of similarity/dissimilarity we utilized two established methods of estimating profile “fit” or correspondence [1], namely, the sum of absolute difference scores (herein difference scores) and profile correlations (herein correlations). Distance scores provide shape-based estimates of correspondence with higher values indicating poorer correspondence; in contrast, the correlational method estimates correspondence based upon the relative standing of different dimensions – with higher values indicating closer correspondence. As indicated in Table S1 and S2, Stereotypical profiles for both those employed in, and aspiring to, CS occupations exhibited a relatively close correspondence with the O*NET estimate of CS interests in terms of correlations and distance scores. This supports our interpretation of these profiles. Interestingly, we note that Artistic profiles for both those employed in, and aspiring to, CS occupations exhibited relatively poor correspondence with the O*NET estimates of CS interests.

**Comparisons of latent profiles to O*NET estimates of latent profile interest levels**

Within the main manuscript, comparison was made between latent profile scores of individuals employed in, or aspiring to, CS occupations and overall estimates of CS interests based upon O*NET interest scores across all CS occupations. However, we note that the occupation makeup of latent profiles varied. To account for the possibility that our results might be due to the differential occupational composition of latent profiles, we calculated seven separate O*NET estimates of interests based upon the unique occupational makeup of each of the four profiles among those employed in CS occupations and the three profiles among those aspiring to CS occupations. As with the overall O*NET estimate of CS interests, we weighted each of the seven O*NET interest profile estimates according to the percentage occupational representation of persons composing each latent profile. We then compared these O*NET interest estimates with our original latent profile interest scores (see Figs S1 and S2). Overall, patterns across these seven comparisons were consistent with our main findings. Specifically, in all but one case (that of the Uninterested profile for those aspiring to CS occupations), Social interests from latent profiles were higher than those calculated based upon O*NET estimates. In addition, for the Artistic profiles among those employed in and aspiring to CS occupations, levels of Artistic interest were higher than that predicted by O*NET estimates.

**Comparisons of latent profiles found in sub-sample to those found in the broader data set**

We conducted LPAs for both the entire available samples and random sub-samples of 500 responses. For simplicity of interpretation, we present our random sub-samples in the main manuscript. Here, we compare the profiles resulting from these random sub-samples to the profiles resulting from the full samples.

First, we note that occupations from the broader sample were well represented in the smaller sample. Table S3 provides list of the occupations from the larger sample. This table can be compared to Table 2 in the main manuscript. Only one occupation (Electronic Drafters) was not included in the random sample. The O*NET estimate of CS interests for the broader sample was highly correlated with the O*NET estimate of CS interests for the smaller sample (*r* = .99).

As can be seen in Figure S3, ten distinct profiles were found for the larger sample of employed computer scientists (4,059 responses). Of these ten profiles, four profiles resembled those found in the smaller sample of employed computer scientists (i.e., Artistic, Multi-interested, Uninterested, and Stereotypical). The Artistic profile found in the random sub-sample was visually similar to Profile 1 with very high interests in artistic tasks. It also had a relatively high correlation and low distance score comparisons to Profile 1(*r* =.95; *Dist.* = 2.94). As with the Artistic profile, Profile 1 was also mostly composed of responses from women (see Table S4). The Multi-Interested profile found in the smaller sample was visually similar to Profile 3 with high interests in all tasks. It also had a relatively high correlation and low distance score comparisons to Profile 3 (*r* = .88; *Dist.* = 2.20). The Uninterested profile found in the smaller sample visually resembled Profile 8 with relatively low interests in all tasks and relatively high correlation and low distance score comparisons (*r* = .86; *Dist.* = 2.57). Finally, the Stereotypical profile found in the smaller sample was visually similar to Profile 5 with high interests in realistic tasks and low interests in artistic tasks and with relatively high correlation and low distance score comparisons (*r* = .87; *Dist.* = 3.76).

As can be seen in Figure S4, six distinct profiles for the larger sample of aspiring computer scientists (603 responses). Of these six profiles, half of them resembled the interest profiles found in the smaller sample of aspiring computer scientists (i.e., Artistic, Uninterested, and Stereotypical). The Artistic profile found in the smaller sample was similar to Profile 6 with high interests in artistic tasks. It also had a relatively high correlation and low distance score comparisons to Profile 6 (*r* = .97; *Dist.* = 6.17). Both profiles were again mostly made up of women (see Table S5). The Uninterested profile found in the smaller sample resembled Profile 2 with low interests in all tasks. It also had a relatively high correlation and low distance score comparisons to Profile 2 (*r* = .79; *Dist.=*1.27). And the Stereotypical profile found in the smaller sample was like Profile 1 with low interests in artistic tasks and relatively high correlation and low distance score comparisons (*r* = .91; *Dist.* = 1.58).

Finally, we note that profiles found in the broader sample show similar patterns to those found in the smaller sample. As indicated in Tables S4 and S5, Stereotypical profiles identified in the broader sample exhibited a relatively close correspondence with the O*NET estimate of CS interests in terms of correlations and distance scores. The Stereotypical profile found in the employed data set had a relatively high correlation and low distance score comparisons to O*NET (r = .73; Dis. = 4.08). The Stereotypical profile found in the aspiring CS data set also had a relatively high correlation and low distance score comparisons to O*NET (r =. 62, Dis. = 4.86). Artistic profiles identified in the broader sample also exhibited relatively poor correspondence with the O*NET estimates of CS interests. The Artistic profile found in the employed data set had a negative correlation and high distance score comparisons to O*NET (r = -.34, Dis. = 10.52). The Artistic profile found in the aspiring CS sample also had a negative correlation and high distance score comparison to O*NET (r = -.19, Dis. = 7.70). These findings are consistent with was found in the smaller sample and reported in the main manuscript.

|  |  | Average Interest Dimension Score | | | | | |  | O*NET comparison | |
| --- | --- | --- | --- | --- | --- | --- | --- | --- | --- | --- |
| Profile | Responses | R | I | A | S | E | C |  | D | r |
| Artistic | 99 | 1.79 | 3.85 | 5.34 | 3.7 | 3.51 | 2.58 |  | 10.52 | -0.34 |
| (72% women) |  |  |  |  |  |  |  |  |  |  |
| Multi-interested | 96 | 4.64 | 4.53 | 5.49 | 3.85 | 3.87 | 3.82 |  | 7.59 | -0.14 |
| (27% women) |  |  |  |  |  |  |  |  |  |  |
| Uninterested | 153 | 1.61 | 2.68 | 1.84 | 2.67 | 3.11 | 2.85 |  | 9.09 | 0.30 |
| (52% women) |  |  |  |  |  |  |  |  |  |  |
| Stereotypical | 152 | 4.21 | 3.89 | 2.72 | 3.2 | 3.81 | 4.18 |  | 4.08 | 0.73 |
| (30% women) |  |  |  |  |  |  |  |  |  |  |
| O*NET CS Interests | - | 3.69 | 5.27 | 2.65 | 1.75 | 3.95 | 4.72 |  | - | - |
| U.S. occupational average | - | 4.73 | 3.45 | 2.17 | 2.84 | 3.49 | 4.16 |  | - | - |

**Table S1**. Interest Profile Scores of Employed Computer Scientists (500 responses) Compared to O*NET Estimates of CS Interests

(R=Realistic, I=Investigative, A=Artistic, S=Social, E=Enterprising, C=Conventional, D=distance scores; *r*=correlations). Interests were assessed in both the online career interest survey and O*NET according to a scale from 1 to 7 with 7 indicating a stronger preference for / a greater relevance of that interest to the occupation.

|  |  | Average Interest Dimension Score | | | | | |  | O*NET comparison | |
| --- | --- | --- | --- | --- | --- | --- | --- | --- | --- | --- |
| Profile | Responses | R | I | A | S | E | C |  | D | r |
| Stereotypical | 181 | 3.27 | 3.73 | 2.14 | 3.2 | 4.44 | 4.27 |  | 4.86 | 0.62 |
| (36% women) |  |  |  |  |  |  |  |  |  |  |
| Uninterested | 115 | 2.17 | 2.7 | 1.6 | 2.04 | 1.91 | 2.77 |  | 9.41 | 0.75 |
| (44% women) |  |  |  |  |  |  |  |  |  |  |
| Artistic | 204 | 3.05 | 4.05 | 5.18 | 3.56 | 3.62 | 3.53 |  | 7.7 | -0.19 |
| (60% women) |  |  |  |  |  |  |  |  |  |  |
| O*NET CS Interests | - | 3.69 | 5.27 | 2.65 | 1.75 | 3.95 | 4.72 |  | - | - |
| U.S. occupational average | - | 4.73 | 3.45 | 2.17 | 2.84 | 3.49 | 4.16 |  | - | - |

**Table S2**. Interest Profile Scores of Aspiring Computer Scientists (500 responses) Compared to O*NET Estimates of CS Interests

(R=Realistic, I=Investigative, A=Artistic, S=Social, E=Enterprising, C=Conventional, D=distance scores; r=correlation). Interests were assessed in both the online career interest survey and O*NET according to a scale from 1 to 7 with 7 indicating a stronger preference for / a greater relevance of that interest to the occupation.

**
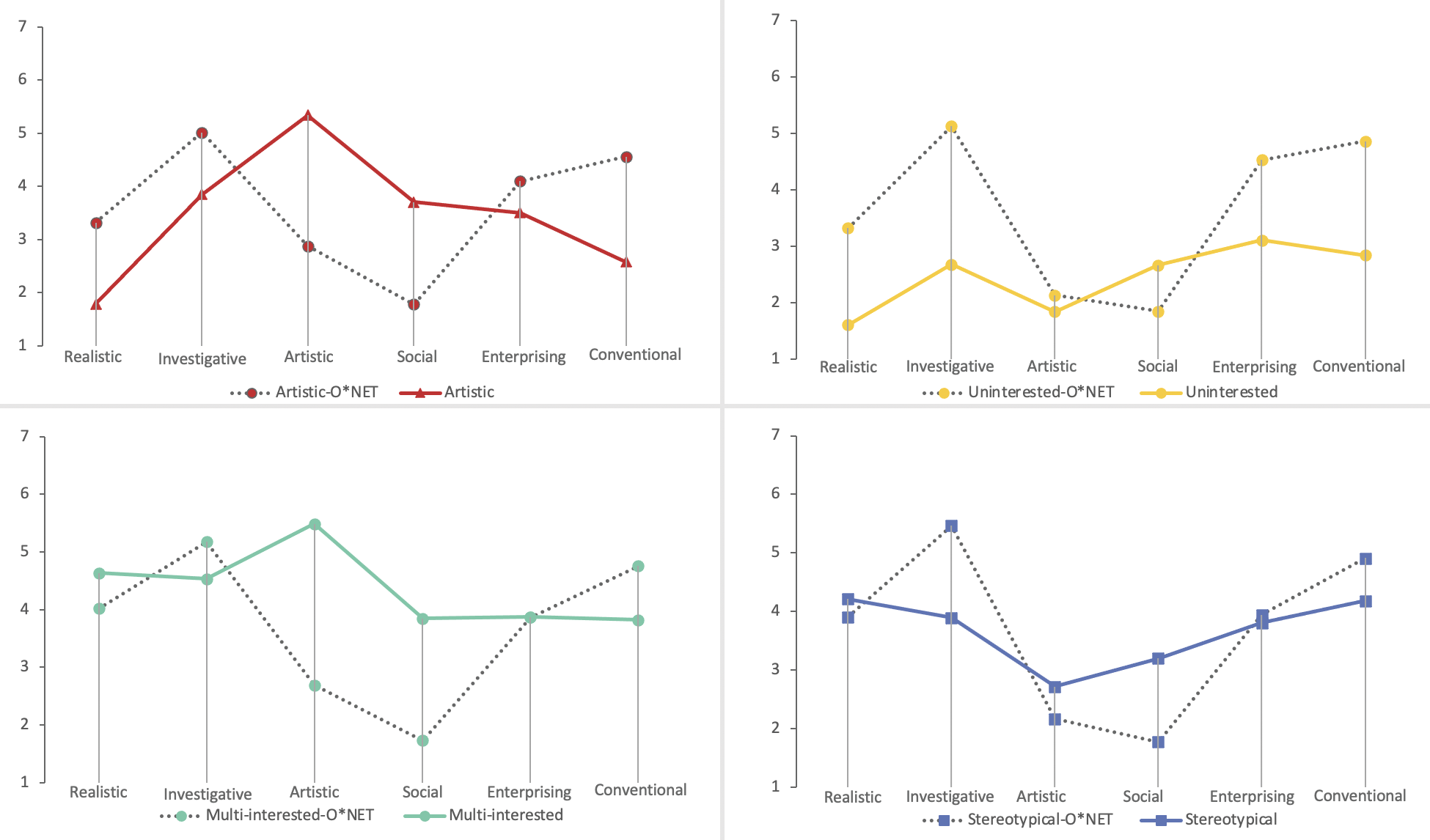
**

**Figure S1.**  Interest Profiles of Employed Computer Scientists (500 responses) Compared to O*NET Estimate of Profile Interests.

**
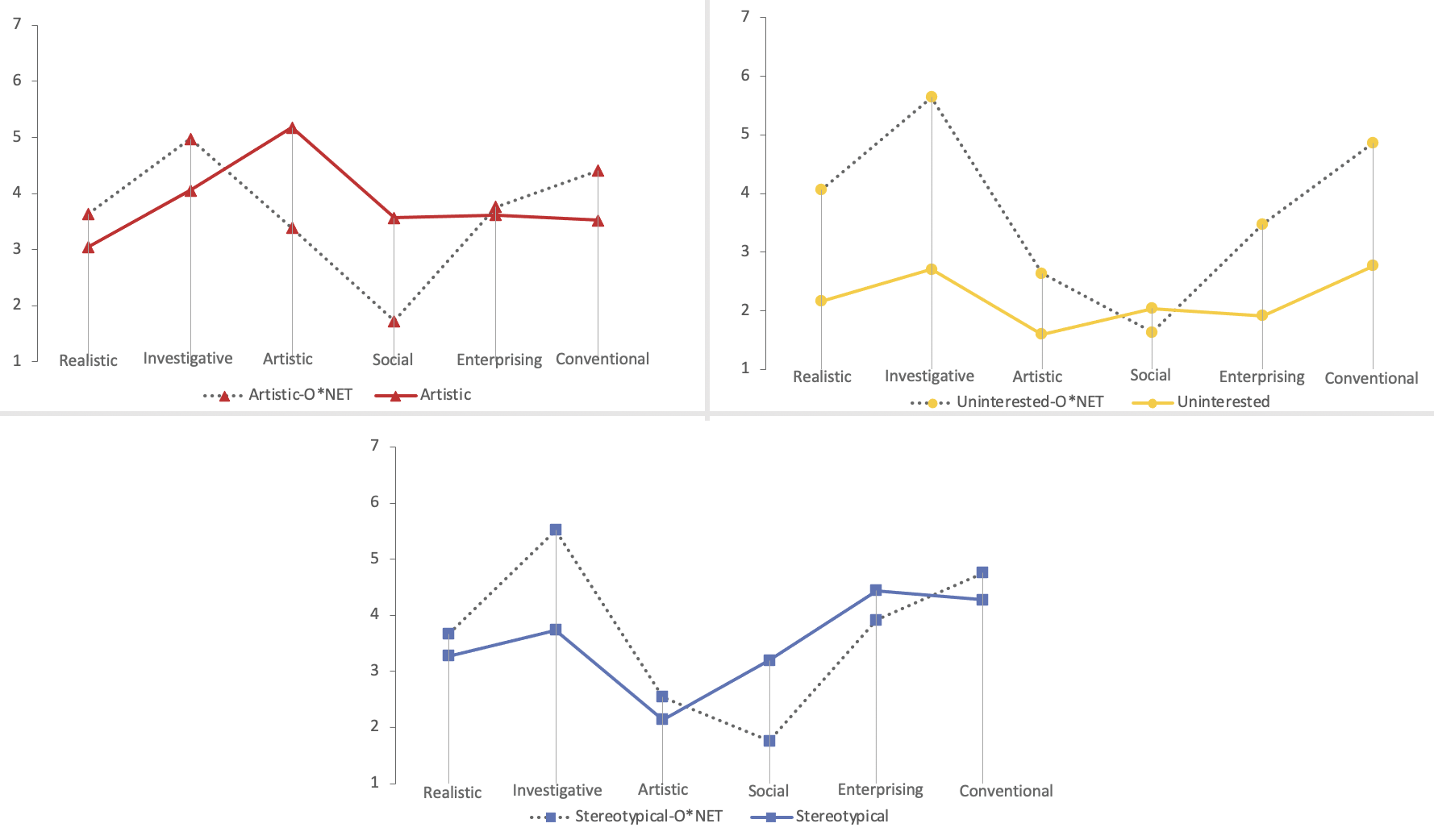
**

**Figure S2.**  Interest Profiles of Aspiring Computer Scientists (500 responses) Compared to O*NET Estimate of Profile Interests.

**Table S3.** Job Titles Characterized as CS and Included in the Full Sample (4,662 responses)

| **Job Titles** | **Count of Responses** |
| --- | --- |
| \| Market Research Analysts and Marketing Specialists \| \| --- \| \| Software Developers, Applications \| \| Information Technology Project Managers \| \| Computer and Information Systems Managers \| \| Computer Systems Analysts \| \| Graphic Designers \| \| Operations Research Analysts \| \| Computer User Support Specialists \| \| Web Developers \| \| Computer Programmers \| \| Software Developers, Systems Software \| \| Business Intelligence Analysts \| \| Network and Computer Systems Administrators \| \| Search Marketing Strategists \| \| Computer and Information Research Scientists \| \| Information Security Analysts \| \| Computer Hardware Engineers \| \| Database Administrators \| \| Software Quality Assurance Engineers and Testers \| \| Computer Network Architects \| \| Quality Control Analysts \| \| Intelligence Analysts \| \| Database Architects \| \| Computer Systems Engineers/Architects \| \| Air Traffic Controllers \| \| Data Entry Keyers \| \| Desktop Publishers \| \| Computer Network Support Specialists \| \| Securities and Commodities Traders \| \| Logistics Managers \| \| Logistics Analysts \| \| Computer, Automated Teller, and Office Machine Repairers \| \| Quality Control Systems Managers \| \| Sound Engineering Technicians \| \| Computer Science Teachers, Postsecondary \| \| Video Game Designers \| \| Financial Quantitative Analysts \| \| Geographic Information Systems Technicians \| \| Audio-Visual and Multimedia Collections Specialists \| \| Web Administrators \| \| Clinical Data Managers \| \| Data Warehousing Specialists \| \| Computer Numerically Controlled Machine Tool Programmers \| \| Gaming Supervisors \| \| Microsystems Engineers \| \| Electronic Drafters \| \| Robotics Technicians \| | \| 581 \| \| --- \| \| 429 \| \| 400 \| \| 288 \| \| 275 \| \| 273 \| \| 227 \| \| 201 \| \| 169 \| \| 153 \| \| 148 \| \| 144 \| \| 137 \| \| 104 \| \| 102 \| \| 81 \| \| 77 \| \| 64 \| \| 60 \| \| 58 \| \| 58 \| \| 57 \| \| 53 \| \| 52 \| \| 50 \| \| 48 \| \| 44 \| \| 42 \| \| 42 \| \| 34 \| \| 25 \| \| 24 \| \| 24 \| \| 23 \| \| 20 \| \| 16 \| \| 14 \| \| 13 \| \| 12 \| \| 12 \| \| 8 \| \| 7 \| \| 5 \| \| 4 \| \| 2 \| \| 1 \| \| 1 \| |

**Figure S3.** Interest Profiles from larger sample of Those Employed in CS (4,059 responses) Compared to O*NET Estimations of CS Interests. Interests were assessed in both the online career interest survey and O*NET according to a scale from 1 to 7, with 7 indicating a stronger preference for / a greater relevance of that interest to the occupation.

|  |  | Average Interest Dimension Score | | | | | |  | O*NET comparison | |
| --- | --- | --- | --- | --- | --- | --- | --- | --- | --- | --- |
| Profile | Responses | R | I | A | S | E | C |  | D | r |
| Profile 1/Artistic (66% women) | 384 | 1.54 | 3.70 | 5.24 | 3.04 | 2.23 | 2.09 |  | 11.66 | -0.32 |
| Profile 2  (39% women) | 435 | 4.00 | 4.50 | 5.25 | 3.17 | 2.76 | 3.67 |  | 7.57 | -0.02 |
| Profile 3/Multi-interested (36% women) | 439 | 5.13 | 4.42 | 5.52 | 4.21 | 4.63 | 4.28 |  | 8.88 | -0.30 |
| Profile 4  (64% women) | 457 | 1.79 | 2.38 | 1.82 | 3.24 | 4.45 | 3.11 |  | 8.21 | 0.14 |
| Profile 5/Stereotypical (20% women) | 251 | 4.71 | 3.62 | 1.98 | 2.42 | 2.51 | 4.00 |  | 5.79 | 0.56 |
| Profile 6 (20% women) | 149 | 5.69 | 3.58 | 2.08 | 3.43 | 5.08 | 4.83 |  | 7.11 | 0.45 |
| Profile 7  (51% women) | 177 | 2.02 | 5.27 | 2.03 | 2.92 | 3.31 | 3.86 |  | 4.45 | 0.75 |
| Profile 8/Uninterested (54% women) | 563 | 1.55 | 2.41 | 1.67 | 2.15 | 2.10 | 2.32 |  | 9.74 | 0.52 |
| Profile 9 (35% women) | 645 | 3.78 | 3.95 | 2.98 | 3.36 | 4.19 | 4.16 |  | 4.29 | 0.82 |
| Profile 10  (73% women) | 559 | 2.06 | 4.07 | 5.23 | 4.10 | 4.49 | 2.90 |  | 9.73 | -0.30 |
| O*NET CS Interests | - | 3.43 | 5.07 | 2.35 | 1.73 | 4.00 | 4.52 |  |  |  |
| U.S. occupational average | - | 4.73 | 3.45 | 2.17 | 2.84 | 3.49 | 4.16 |  | - | - |

**Table S4.** Interest Profile Scores of broader sample of Employed Computer Scientists (4,059 responses) Compared to O*NET Estimates of CS Interests (R=Realistic, I=Investigative, A=Artistic, S=Social, E=Enterprising, C=Conventional, D=distance scores; r=correlation). Interests were assessed in both the online career interest survey and O*NET according to a scale from 1 to 7 with 7 indicating a stronger preference for / a greater relevance of that interest to the occupation.

**Figure S4.** Interest Profiles from larger sample of Those Aspiring to CS (603 responses) Compared to O*NET Estimations of CS Interests. Interests were assessed in both the online career interest survey and O*NET according to a scale from 1 to 7, with 7 indicating a stronger preference for / a greater relevance of that interest to the occupation.

|  |  | Average Interest Dimension Score | | | | | |  | O*NET comparison | |
| --- | --- | --- | --- | --- | --- | --- | --- | --- | --- | --- |
| Profile | N | R | I | A | S | E | C |  | D | r |
| Profile 1/Stereotypical (46% women) | 113 | 2.27 | 3.48 | 2.09 | 3.20 | 4.65 | 4.21 |  | 5.44 | 0.54 |
| Profile 2/Uninterested (49% women) | 148 | 1.79 | 2.67 | 1.71 | 2.08 | 2.25 | 2.41 |  | 8.89 | 0.76 |
| Profile 3  (33% women) | 131 | 4.48 | 4.48 | 4.87 | 3.78 | 4.03 | 4.47 |  | 6.30 | 0.23 |
| Profile 4  (15% women) | 78 | 5.09 | 4.24 | 1.85 | 2.89 | 3.71 | 5.08 |  | 4.99 | 0.70 |
| Profile 5  (83% women) | 80 | 2.04 | 3.98 | 5.33 | 4.55 | 4.09 | 3.42 |  | 9.47 | -0.41 |
| Profile 6/Artistic (77% women) | 53 | 1.77 | 3.55 | 5.03 | 2.25 | 2.34 | 1.89 |  | 10.67 | -0.19 |
| O*NET CS Interests | - | 3.43 | 5.07 | 2.35 | 1.73 | 4.00 | 4.52 |  | - | - |
| U.S. occupational average | - | 4.73 | 3.45 | 2.17 | 2.84 | 3.49 | 4.16 |  | - | - |

**Table S5.** Interest Profile Scores of broader sample of Aspiring Computer Scientists (603 responses) Compared to O*NET Estimates of CS Interests (R=Realistic, I=Investigative, A=Artistic, S=Social, E=Enterprising, C=Conventional, D=distance scores; r=correlation). Interests were assessed in both the online career interest survey and O*NET according to a scale from 1 to 7 with 7 indicating a stronger preference for / a greater relevance of that interest to the occupation.

**References**

1. Su, R., Murdock, C., & Rounds, J. *Person-environment fit.* (2015).
2. Spurk, D., Hirschi, A. Wang, M., Valero,D.. & Kauffeld, S. Latent profile analysis: a review and “how to”guide of its application within vocational behavior research. Journal of Vocational Behavior. (2020).
